# Acceptability of HIV counselling and testing among tuberculosis patients in south Ethiopia

**DOI:** 10.1186/1472-698X-7-4

**Published:** 2007-05-30

**Authors:** Degu Jerene, Aschalew Endale, Bernt Lindtjørn

**Affiliations:** 1 Centre for International Health, University of Bergen, Norway; 2 Arba Minch Hospital, Ethiopia

## Abstract

**Background:**

To benefit from available care and treatment options, patients should first be counselled and tested for HIV. Our aim was to assess the acceptability of HIV testing among tuberculosis patients under routine care conditions in south Ethiopia.

**Methods:**

We interviewed all adult tuberculosis patients who were treated at Arba Minch Hospital in Ethiopia between January and August 2005. After recording socio-demographic information and tuberculosis treatment history, we referred those patients who showed initial willingness to a counsellor for HIV counselling and testing. Rapid test methods were used following a pretest counselling session. The results were disclosed during a post-test counselling session. We used the logistic regression method to assess factors associated with willingness and acceptability.

**Results:**

190 adult tuberculosis patients were treated at the hospital and all of them consented to take part in the study. Their median age was 30 years (range, 15–68) and 52% of them were males. 49 patients (26%) were previously tested including 29 (59%) HIV positive. Of 161 patients (excluding the 29 already positive), 118 (73%) were willing to be tested and 58% (68/118) of those willing accepted the test. The overall acceptability rate was 35% (56/161). Fourteen (20.6%) were HIV positive and women were more likely to be HIV infected (p = 0.029). Unemployment and self-perceived high risk of HIV infection were associated with initial willingness (OR [95%CI]:2.6 [1.3–5.5] vs. 5.0 [1.1–22.4], respectively). However, only being unemployed was associated with accepting the test (OR = 4.2; 95%CI = 1.9–9.3).

**Conclusion:**

The low acceptability of HIV counselling and testing among tuberculosis patients poses a challenge to the scale-up of TB/HIV collaborative efforts. There is a need for alternative counselling and testing strategies.

## Background

Voluntary counselling and testing (VCT) for human immunodeficiency virus (HIV) has been carried out in many places with excellent results, it is cost-effective, and a gateway to most HIV related services including provision of antiretroviral drugs [1,2]. However, in most sub-Saharan African countries, many people still do not know their HIV status [3]. Some treatment programmes have reported high early mortality in patients receiving antiretroviral therapy because of late presentation [4]. Therefore, early detection of HIV infection is not only useful in preventing further infection but also part of the strategy to improve treatment outcomes.

This cannot be achieved through the traditional VCT alone and different alternatives have been proposed One such approach is routine counselling and testing of patients, also called provider-initiated counselling and testing [5,6]. In this approach, the basic conditions of confidentiality, consent and counselling apply and the standard pre-test counselling used in VCT is adapted to ensure informed consent, without a full education and counselling session. To be able to provide informed consent, patients need to know: (i) the clinical and prevention benefits of testing; (ii) the right to refuse; (iii) the follow-up services that will be offered; and (iv) the importance of sharing results with a partner in case of positive results [6].

Tuberculosis (TB) patients are one of the target populations for the provider-initiated counselling and testing [5-7]. The WHO has, therefore, incorporated routine counselling and testing as a component of TB/HIV collaborative efforts [8]. Subsequently, the National TB and HIV guideline in Ethiopia recommends HIV counselling and testing as a routine care for TB patients [9]. However, the acceptability of this approach has not been studied. Assessments done on the acceptability of VCT among patients and in the general population gave mixed results [10-12]. The objective of this study was to assess the acceptability of HIV counselling and testing among TB patients under routine care conditions in southern Ethiopia.

## Methods

### Study setting

Arba Minch hospital (AMH) has a catchment population of 1.5 million. This public district hospital is in the Gamo Goffa Zone, 500 km south of Addis Ababa. The hospital started Directly Observed Treatment, Short Course (DOTS) for TB in 1996. Diagnosis of pulmonary tuberculosis is made using a combination of clinical examination, Acid-Fast Bacilli (AFB) staining and/or chest x-ray. Doctors are responsible for diagnosing smear negative and extra pulmonary cases, while smear positive cases are also treated by trained nurses. The hospital follows the National Tuberculosis and Leprosy Control manual of Ethiopia [13].

The HIV unit, coordinated by a doctor, provides VCT, treatment for opportunistic diseases and antiretroviral therapy (ART). There are five counsellors, two laboratory technicians, two community agents and a data clerk working with the HIV unit. The counsellors are nurses with extra training on counselling and testing. They refer all HIV positive patients to the treating doctor who examines, stages according to the WHO clinical staging and starts treatment for opportunistic infections and begins ART as appropriate. Community agents provide home based support and counselling.

At the time of the study, there was only one stand-alone VCT centre at Arba Minch town. Clients who test positive are referred to AMH for medical care.

### Patients

All consecutive adult patients (age more than 15 years) with tuberculosis, (both existing and newly diagnosed) visiting the hospital between January and August 2005 made up the study population.

### Data collection

We used a questionnaire as a data collection tool. First, the nurse at the TB clinic asked details of socio-demographic information (age, gender, marital status, occupation, religion, monthly income, education, address); previous history of tuberculosis including the date, type and phase of current treatment; history of HIV testing including test results; and whether the patient was on follow-up for HIV related condition. To assess their HIV related risk we asked; "How do you rate your HIV related risk?" They were given "High" or "Low" as a choice. We classified employment status as "Employed" and "Unemployed".

Patients were then asked if they would be willing to take HIV counselling and testing if offered. Those who showed willingness (as defined below) were sent to a trained counsellor in a separate room. Patients with previous HIV positive results were not asked to be pretest counselled. Following the pretest counselling, clients were asked to give 5 ml of blood for HIV testing. A trained laboratory technician did the HIV test in a separate room.

We used the rapid HIV testing algorithm according to the national protocol [14]. Therefore, we used Determine (ABOTT JAPAN CO.LTD, Tokyo, Japan) as the screening test followed by Capillus (Trinity Biotec Plc, Wicklow, Ireland). Unigold (Trinity Biotec Plc, Wicklow, Ireland) was used as tie-breaker test in case of inconclusive results. The patients were told about the results on the same day or within the shortest convenient time during a post-test counselling session. Those who were HIV positive were offered the chance to have clinical follow-up at the hospital. If the client did not return for the results within 2 weeks of the appointment date, it was considered non-acceptability of the post-test counselling.

### Ethical considerations

The Regional Committee for Medical Research Ethics in Western Norway and the National Ethics Review Committee in Ethiopia approved the study protocol. The study participants gave informed written consent for taking part in the study and for HIV testing.

### Definitions

Because of lack of standard terminology, we used the following definitions. **Willingness **was assessed using the question "Do you want to be counselled for HIV testing?" Those who replied "Yes" were labelled as "willing". Acceptability of pretest counselling and testing was evaluated if they agreed to be counselled and tested. Thus, the **pre-test counselling acceptability rate **was defined as the number of patients counselled and tested divided by the total willing. Similarly, we defined the **post-test counselling acceptability rate **as those who received the results for those who were counselled and tested. **Overall acceptability rate **was defined as the total number of patients who received the results out of the total interviewed for willingness.

### Statistical methods

We used acceptability as the main outcome variable. We evaluated acceptability in three stages: (i) showed willingness for pretest counselling, (ii) pretest counselled and HIV tested, and (iii) post-test counselled and received the result.

Using the Logistic Regression method, we assessed potential factors associated with acceptability of HIV testing. In the regression model, we included information on age, gender, occupation, education, address, phase of TB treatment (intensive phase vs. continuation phase), and self-perceived HIV risk (low vs. high) as possible predictors. We selected these variables based on a review of the literature and on our own hypothesis. In a univariate analysis, we tested each predictor variable separately and then we included those variables with P-values between 0.05 and 0.10 into a multivariate model. Those predictor variables in the multivariate model of "willingness to be pre-test counselled" were forced into the multivariate model of "acceptability of pretest counselling" irrespective of the P-values in the univariate analysis of the later model. This was to see whether the same factors affected both willingness and acceptability.

We present the results as Odds Ratios (OR) with 95% confidence interval (95% CI). Both the Omnibus Tests of Model Coefficients (If P < 0.05, the model is considered good) and the Hosmer and Lemeshow Test (If P > 0.05, the model is considered good) were used to describe the performance of each model. SPSS version 13.0 was used for data analysis.

## Results

### Patient characteristics

Between January and August 2005, 190 adult TB patients received treatment at the hospital. 52% (98) of the patients were men, and the median age of the study participants was 30 years (range, 15–68). 102 (54%) patients came from rural areas and about three-quarter (74%) had monthly income below $ 20 USD. 45% (102/190 patients) were unemployed. Twenty-eight of 190 patients (15%) gave previous history of at least one episode of tuberculosis and in 26 of these, the episode was in the last five years. At the interview, 40% (78/190), 36% (68/190) and 23% (44/190) of the patients were on treatment for sputum positive pulmonary tuberculosis (PTB+), extra pulmonary tuberculosis (EPTB), and smear negative pulmonary tuberculosis (PTB-), respectively. Table 1 shows patient characteristics.

**Table 1:** Some patient characteristics, Arba Minch Hospital, 2006 (n = 190)

Characteristic	Frequency	Percentage
Gender		
Men	98	51.6
Women	92	48.4
Age (years)		
15–24	57	30.0
25–34	65	34.2
35–44	46	24.2
45+	22	11.6
Employment status		
Unemployed	104	54.7
Employed	86	45.3
Area of residence		
Rural	102	53.7
Urban	88	46.3
Education		
Illiterate	99	52.1
Primary education	53	27.9
Secondary and above	38	20.0
TB diagnosis		
PTB+	78	41.1
EPTB	68	35.8
PTB-	44	23.2
Phase of treatment		
Intensive	102	53.7
Continuation	88	46.3

### Previous HIV diagnosis and treatment

Of 190 patients, 49 (26%) were previously HIV tested (27 men and 22 women). 42% (37 of 88 patients) and 12% (12/102) of urban and rural residents, respectively reported previous history of testing (χ^2 ^= 22.6, P < 0.001). 59% (29/49 patients) of those previously tested were HIV positive, 33% (6/49) were HIV negative and 8% (4/49) did not know their result. HIV prevalence was higher among the urban residents (70% [26/37]) than among patients from the rural areas (25% [3/12]) (χ^2 ^= 7.7, df = 2, p = 0.021). The HIV prevalence rate was higher among women than men but this was not statistically significant (men 52% vs. women 68%, χ^2 ^= 4.6, p = 0.102). 86% (25/29) of the HIV positive patients were already registered with the HIV unit for clinical follow up. 14 of the 25 patients (56%) received cotrimoxazole prophylaxis, 20% (5/25) antiretroviral therapy and one patient received both cotrimoxazole prophylaxis and antiretroviral therapy.

### Perceived HIV risk and awareness

Over a quarter of the patients (50/190) perceived themselves as being at high-risk of HIV infection. The same number of patients (50/190) rated their HIV-related awareness as 'poor'. Only 11% (21/190) of the patients said they had "very good" knowledge about HIV and 62% (119/190) said they had 'good' knowledge.

### Willingness and acceptability of HIV testing

**Figure 1:**
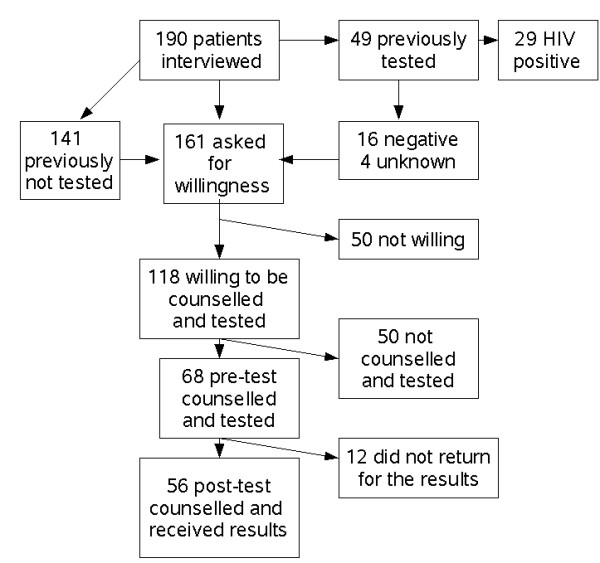
Acceptability of HIV counseling and testing at the different phases of the counselling and testing process, Arba Minch Hospital, 2006.

Of 161 patients (excluding 29 patients with previously confirmed HIV positive results), 118 (73%) were willing to be counselled and tested. Nevertheless, only 58% (68/118) of those willing were counselled and tested. Fifty-six of those tested attended the post-test counselling session making the post-test counselling acceptability rate 82% (56/68). Thus, the overall acceptability rate was 35% (56/161) [Figure 1].

Fourteen patients of those tested were HIV infected, making the HIV prevalence rate 20.6% (14/68) among the newly tested. The HIV and TB co-infection rate was higher among women (32.3% versus 10.8% among men; χ^2 ^= 4.7; p = 0.029). The prevalence among rural and urban residents was similar (16.3% rural versus 28.0% urban; χ^2 ^= 1.3; p = 0.249). The combined previous (29/49) and current (14/68) HIV infection rate was 36.7% (43/117).

On logistic regression analysis, self-perceived high-risk of being HIV infected was associated with initial willingness to be tested (OR [95%CI] = 5.0 [1.1–22.4]; p = 0.036) but it was not associated with actually being counselled and tested (OR [95%CI] = 0.6 [0.2–1.5, p = 0.266]. On the other hand, unemployed patients were more likely to be both willing (OR [95%CI = 2.6 [1.1–5.5]; p = 0.010) and accepting the test (OR [95%CI] = 3.7 [1.6–8.6]; p = 0.002). Both willingness and accepting the test were not affected by age, gender, education, marital status of the patient, or phase of TB treatment (P > 0.1 in all). Tables 2 and 3 show the results of the logistic regression analyses.

**Table 2:** Logistic regression analysis of odds ratio (OR) for willingness in relation to potential predictors, Arba Minch Hospital, 2006 (n = 161).

Predictor variable	Univariate model		Multivariate model	
	
	OR (95%CI)	P-value	OR (95%CI)	P-value
Employment status				
Employed	1.0		1.0	
Unemployed	2.7 (1.3–5.6)	0.007	2.6 (1.3–5.5)	0.010
Perceived HIV risk				
Low	1.0		1.0	
High	5.2 (1.2–23.2)	0.029	5.0 (1.1–22.4)	0.036
Gender				
Women	1.0			
Men	1.4 (0.7–2.7)	0.386	-	
Educational status				
Primary and above	1.0			
Illiterate	1.5 (0.7–3.0)	0.277	-	
Area of residence				
Rural	1.0			
Urban	1.1 (0.5–2.2)	0.872	-	
Age (years)				
>= 35	1.0			
15–34	1.4 (0.7–2.8)	0.386	-	
Phase of treatment				
Intensive	1.0			
Continuation	1.5 (0.7–2.9)	0.290	-	

**Multivariate model**: Occupation and HIV risk (Omnibus test, χ^2 ^= 13.8, p = 0.001; HL test, χ^2 ^= 3.5, p = 0.172)

**Univariate model**: Occupation (Omnibus test, χ^2 ^= 7.6, p = 0.006; HL test, χ^2 ^= 0; HIV risk (Omnibus test, χ^2 ^= 7.0, p = 0.008; HL test, χ^2 ^= 0)

**Table 3:** Logistic regression analysis of odds ratio (OR) for acceptability of pretest counselling in relation to potential predictors, Arba Minch, October 2006 (n = 118)

Predictor variable	Univariate model		Multivariate model	
	
	OR (95%CI)	P-value	OR (95%CI)	P-value
Perceived HIV risk				
Low	1.0			
High	0.6 (0.2–1.5)	0.299	0.6 (0.2–1.5)	0.266
Employment status				
Employed	1.0		1.0	
Unemployed	4.2 (1.9–9.3)	0.000	3.7 (1.6–8.6)	0.002
Gender				
Women	1.0			
men	1.1 (0.5–2.2)	0.636	-	
Educational status				
Primary and above	1.0			
Illiterate	2.1 (1.0–4.5)	0.060	1.7 (0.7–3.8)	0.227
Address				
Rural	1.0			
Urban	0.9 (0.4–2.0)	0.842	-	
Age (years)				
>= 35	1.0			
15–34	0.9 (0.4–1.9)	0.665	-	
Phase of treatment				
Intensive	1.0			
Continuation	1.3 (0.5–2.9)	0.469	-	

## Discussion

Despite the high rate (73%) of initial willingness, only a third (35%) of tuberculosis patients were counselled and tested. Patient's self-perception of HIV risk was an indicator of initial willingness but not of accepting the test. On the other hand, unemployed patients were consistent in being both willing to be tested and accepting the test. About a quarter of our patients had been tested before, and two-thirds of the HIV positive patients were among these patients.

The low acceptability of HIV counselling and testing among TB patients represents a challenge to the scale-up of TB/HIV collaborative efforts and highlights the need for identifying and removing the underlying causes. Patient-related factors such as self-perceived risk of HIV infection are important indicators of initial willingness but not of actually accepting the test in some sub-Saharan African populations [11,15]. These behavioural factors explain only part of the reasons for the low acceptability of HIV counselling and testing. Concerns about confidentiality, convenient timing of the service and place of the testing site have been described as determinants of acceptability [16,17].

Studies from Malawi on acceptability of VCT among TB patients [12,18,19] showed that acceptability was above 90% under research conditions but was lower (59%) under routine care conditions [19]. On the other hand, a randomized trial among the general population in Zambia showed that acceptability of VCT was low (11.8%) in a clinic setting but higher (55.8%) in an optional site [11]. They also found discrepancy in the readiness (willingness in our case) and acceptability.

In our study, the pretest counselling and testing acceptability rate of 58% among those who showed initial willingness is comparable to a Malawian study done under routine care conditions [19]. It also compares well to the Zambian finding of 55.8% in the general population at an optional site [11]. In a community-based cohort in rural Uganda, only 62.5% of the participants accepted VCT despite much higher (93%) rate of initial willingness [20].

Patients with felt high risk of HIV infection were about five times more likely to be willing to be tested. In agreement with our results, a study in a prevention of mother-to-child HIV transmission (pMTCT) clinic in Uganda showed higher acceptability rate among those at perceived high risk of HIV infection [15]. Also in Zambia young people with felt high risk of HIV infection were more likely to be willing to be tested [11]. Despite the difference in the populations studied (TB patients, pregnant women, and general population), these studies show that individual's self-perceived risk of HIV infection is an important indicator of willingness to be tested. However, self-perception of HIV risk was not associated with accepting the test, as has been noted by others [11].

The long process of counselling and testing is a likely cause for the low acceptability despite high initial willingness. Doing the testing in the TB clinic itself, for example, could have improved the acceptability rate. This requires training of TB clinic staff in the techniques of HIV testing.

The combined HIV-TB co-infection rate was 37%. This is lower than reports from major urban settings in Ethiopia. In Addis Ababa, 45% of TB patients were co-infected with HIV [21]. In Ethiopia, the adult HIV prevalence is estimated at 4.4% (12.6% urban and 2.6% rural) [22]. Given the predominance of patients from rural areas, the prevalence in our TB patients is about 10-fold higher than that of the general population. This implies that a large number of HIV infected patients may be missed unless there is active search for their infection status

We did this study under routine care conditions. The findings may thus be extrapolated to other similar settings. However, since we did not study reasons related to the infrastructure or personnel related factors, we may have omitted some explanations about the low acceptability of HIV counselling and testing among our TB patients. Also, the small sample size, as evidenced by wide confidence intervals, is an additional limitation of this study. Further studies on the infrastructure or personnel related factors using larger sample from multiple sties could provide complementary information.

## Conclusion

The low acceptability of HIV counselling and testing among tuberculosis patients poses a challenge to the scale-up of TB/HIV collaborative efforts. This highlights the need for alternative and improved ways of identifying more HIV infected patients among the TB patients. One such approach is training the TB clinic staff in the techniques of HIV counselling and testing, and doing the testing at the TB clinic itself.

## Competing interests

The author(s) declare that they have no competing interests.

## Authors' contributions

DJ conceived of and designed the study, supervised data collection, analyzed the data, drafted the paper and approved the final version. AE co-supervised data collection, contributed to the drafting of the paper and approved the final version. BL contributed to the conception, designing, data analysis, drafting and approval of the manuscript.

## Pre-publication history

The pre-publication history for this paper can be accessed here:


